# Regulating Interfacial Li‐Ion Transport via an Integrated Corrugated 3D Skeleton in Solid Composite Electrolyte for All‐Solid‐State Lithium Metal Batteries

**DOI:** 10.1002/advs.202104506

**Published:** 2022-01-17

**Authors:** Rong Fan, Wenchao Liao, Shuangxian Fan, Dazhu Chen, Jiaoning Tang, Yong Yang, Chen Liu

**Affiliations:** ^1^ Shenzhen Key Laboratory of Polymer Science and Technology Guangdong Research Center for Interfacial Engineering of Functional Materials College of Materials Science and Engineering Shenzhen University Shenzhen 518060 China; ^2^ Collaborative Innovation Center of Chemistry for Energy Materials State Key Laboratory for Physical Chemistry of Solid Surface College of Chemistry and Chemical Engineering Xiamen University Xiamen 361005 China

**Keywords:** 3D skeleton, composite electrolytes, interfacial ion conduction, solid‐state batteries

## Abstract

Although solid composite electrolytes show tremendous potential for the practical solid‐state lithium metal batteries, searching for a straightforward tactic to promote the ion conduction at electrolyte/electrode interface, especially settling lithium dendrites formation caused by the concentration gradient polarization, are still long‐standing problems. Here, the authors report a corrugated 3D nanowires‐bulk ceramic‐nanowires (NCN) skeleton reinforced composite electrolyte with regulated interfacial Li‐ion transport behavior. The special and integrated NCN skeleton endows the electrolyte with fast Li‐ion transfer and solves the Li^+^ concentration polarization at electrode/electrolyte interface, thereby eliminating the energy barrier originated from the redistribution of charge carriers and offering homogeneous interfacial Li‐ion flux on lithium anode. As a “double insurance”, the bulk ceramic sheet in 3D framework enables the electrolyte to block the mobility of anions. The rational designed NCN composite electrolyte exhibits excellent ionic conductivity and the assembled all‐solid‐state battery possesses 90.2% capacity retention after 500 cycles. The proposed strategy affords a special insight in designing high‐performance solid composite electrolytes.

## Graphical abstract

A corrugated 3D nanowires‐bulk ceramic‐nanowires skeleton reinforced composite electrolyte is designed with fast Li‐ion transfer kinetics and to solve the Li^+^ concentration polarization at electrode/electrolyte interface, thereby eliminating the energy barrier originated from the redistribution of charge carriers and offering homogeneous interfacial Li‐ion flux on lithium anode.

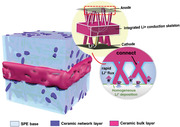

## 1 Introduction

The ever growing needs for portable electronic devices and electric vehicles have dramatically aroused the demand for high‐energy density and safety energy storage systems.^[^ ^1^, ^2^ ^]^ All‐solid‐state lithium metal batteries (ASLMBs) consequently have attracted extensive attention and been deemed as the most promising next‐generation lithium‐ion batteries.^[^ ^3^, ^4^ ^]^ As the key component of ASLMBs, solid‐state‐electrolytes (SSEs) are responsible for transporting lithium ions and isolating the electron conduction between anode and cathode simultaneously. Owing to the high ionic conductivity of ≥10^−3^ S·cm^−1^ have achieved for SSEs to date, especially at elevated temperature, the lithium‐ion conduction ability of SSEs is no longer a major concern.^[^ ^5^, ^6^, ^7^ ^]^ Nevertheless, the performance of all‐solid‐state batteries is still inferior to that of the liquid electrolyte‐based lithium batteries, suggesting that a sluggish transport behavior of lithium ions across electrode/electrolyte interface is at play. The phenomenon is attributed to the following aspects: i) large charge transfer barrier caused by the less effective contact between electrode and solid electrolyte, as well as reduced contact area originated from volume change of electrode during charging/discharging process; ii) the formation of detrimental solid electrolyte interphase (SEI) leads to Li^+^ transfer hindrance; iii) polarization effect at the electrode/electrolyte interface, resulted from the slow and inhomogeneous ion transport in the electrolyte and fast charge transfer reaction from the electrolyte/electrode interface into the electrode.^[^ ^8^ ^–^ ^14^ ^]^

**Figure 1:**
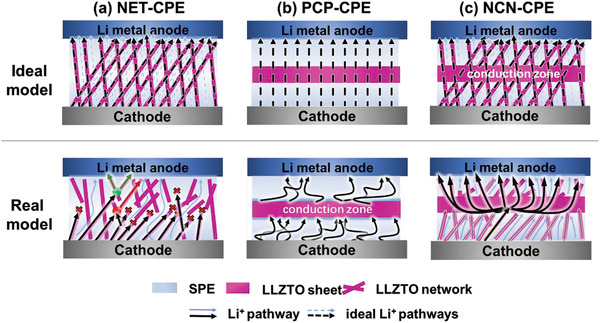
Schematic diagram of lithium‐ion transport in ideal state and practical state in PCP‐CPE, NET‐CPE, and NCN‐CPE.

Over the past few years, important progress has been achieved by interface engineering, including building artificial SEI and lithiophilic interfacial layer to regulate the Li ion deposition on Li anode.^[^ ^15^, ^16^, ^17^, ^18^, ^19^ ^]^ However, those approaches usually require precious control over the chemical reaction conditions with Li anode or choosing appropriate interfacial layer materials. Moreover, the stability of SEI is challenged by the large volume change of electrode during cycling. Thus, searching for a more straightforward strategy via focusing on the design of electrolyte itself is of great importance. Composite polymer electrolytes (CPE) consist of ceramic and polymer components, combining the good interface contact of polymer electrolytes and high ion conductivity as well as high electrochemical stability and mechanical strength of inorganic electrolytes together, are expected to possess good contact with electrodes and adapt their volume change during battery cycling.^[^ ^20^, ^21^, ^22^ ^]^ It has been proved that Li^+^ transport pathways are mainly dominant by the ceramic part in the electrolyte when high loading of ceramic fillers is incorporated.^[^ ^23^, ^24^ ^]^ Thus, manipulating the ceramic component is crucial for homogenizing Li^+^ flux in the composite electrolyte and mechanically inhibiting dendrite growth. It has been well demonstrated and widely reported that introducing inorganic nanowire or 3D structure can effectively provide fast and continuous Li‐ion conduction pathways in the composite electrolytes,^[^ ^25^, ^26^, ^27^, ^28^, ^29^, ^30^, ^31^, ^32^, ^33^, ^34^, ^35^, ^36^, ^37^, ^38^ ^]^ however, the nanowires are easy to agglomerate and most of the delicate porous structures are likely to be collapsed (e.g., the porous structure fabricated via freeze‐drying or spinning method) when compositing with polymer matrix and the rapid ion conduction route would be destructed as shown in the real mode of **Figure** **1**a. Moreover, while solving the problem of uniform ion conduction and good physical interface contact, this kind of structure ignores the energy barrier originated from the redistribution of charge carriers at the electrode/electrolyte interface, which greatly hinders the long stable cycling of the battery. In this regard, Zhou et al.^[^ ^39^ ^]^ built a sandwiched electrolyte with a structure of polymer‐ceramic‐polymer (PCP), which could weaken the electric double layer at electrode/electrolyte interface via the Li^+^ filtration effect of ceramic layer. But the contact of such sandwiched structure with Li anode is dependent on the wettability of polymer layer and could not completely alleviate the adverse effect of the polymer phase since it also contains salt anions (Figure 1b). Additionally, the double electric layer formed at the interface of polymer and ceramic layer is also against the ion conduction.^[^ ^40^ ^]^ Thus, it is necessary to find a way to construct electrolytes with both high ionic conductivity and effective interfacial conduction of Li ions.

Deeply understanding how the structure and composition of SSEs affect the ion transmission both in the SSEs and cross electrolyte–electrode interface is of great significance for achieving a stable and long‐life lithium battery, due to their significant influence on diffusion dynamics and transport pathway of lithium ions for the batteries.^[^ ^41^, ^42^, ^43^, ^44^ ^]^ Here, we design a 3D corrugated garnet skeleton with indented structure, where the nanowire‐network layers are in situ grown on both sides of a bulk ceramic sheet. The special and integrated nanowire‐bulk ceramic‐nanowire (NCN) skeleton, as shown in Figure 1c, could guide a homogeneous and rapid Li^+^ conduction in the electrolyte, which further confines the generation of polarization effect at electrode/electrolyte interface. After infusing with polymer electrolyte in both sides of nanowire networks, low interfacial resistance and good adhesion could be achieved with both electrodes. In the meantime, the garnet ceramic network (NET) reinforced and sandwiched PCP composite electrolytes were also prepared as comparison. The ion transport mechanism at the electrolyte/electrode interface of the designed three structures was analyzed using finite element analysis, and the NCN‐structure ceramic framework can effectively reduce the polarization between electrolyte and electrode via rapid and homogeneous transport of Li ions. The NCN‐structure battery displayed excellent ionic conductivity and stable electrochemical performance in the bending state, as well as long‐term cycling stability. The proposed strategy in this study is expected to understand the underlying mechanism of designing composite electrolytes for long lifespan ASLMBs.

## 2 Results and Discussion

### 2.1 Physiochemical Properties of 3D CPEs

**Figure 2:**
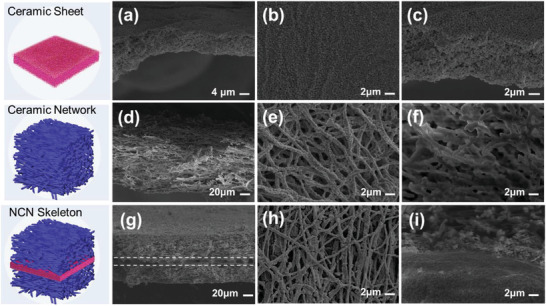
The SEM morphological characterization of the active ceramic skeletons: a–c) LLZTO ceramic bulk prepared by sol‐gel method, d–f) LLZTO nanowire‐network prepared by electrospinning, g–i) sandwiched NCN ceramic skeleton. The first row and the third row exhibit the cross section images and the second row is the top‐view of the ceramic skeleton.

The composite electrolytes employing Li_6.5_La_3_Zr_1.5_Ta_0.5_O_12_ (LLZTO)‐based bulk ceramic, nanowire‐network and integrate nanowire‐bulk ceramic‐nanowire as ceramic skeleton, and polyethylene oxide (PEO) with lithium bis (trifluoromethanesulfonyl)imide (LiTFSI) as solid polymer electrolyte (SPE) matrix are successfully fabricated via a series of sol‐gel method, electrospinning, sintering and casting processes. The designed solid electrolytes are defined as PCP‐CPE, NET‐CPE, and NCN‐CPE, respectively. The morphologies of the active‐ceramic skeletons were characterized by SEM. A bulk and uniform LLZTO ceramic sheet with a thickness of ≈10 µm was obtained from the cross‐section image in **Figure** **2**a–c, demonstrating that sol‐gel method is more conducive to prepare thinner bulk ceramic than that of conventional tablet pressing method. Although the bulk ceramic layer exhibits relatively porous structure, it is still able to offer rapid and homogeneous Li‐ion conduction, as well as sufficient mechanical strength to support the growth of NET network layer.

The electrospun LLZTO nanowire‐network processed with sintering is exhibited in Figure 2d–f, in which the nanowire has a diameter of 200–400 nm. Ideally, the nanowires with Li^+^ conduction ability could offer continuous and rapid Li‐ion pathways, however, the fracture of such 3D network structure can be observed in the enlarged cross‐sectional view (Figure 2f), revealing that the ideal design effect cannot be realized in practical situation. Moreover, these defects would be further aggravated during the infusing process of polymer electrolyte. An integrated and sandwiched ceramic NCN skeleton is shown in Figure 2g–i, which was fabricated by sintering the NET‐ceramic sheet‐NET layers together. The compact integration of nanowire network with ceramic sheet is demonstrated in Figure 2i. Compared with the free‐standing LLZTO network (Figure 2d), a better integrity is kept for the network layer in NCN‐structure. The reason is that the bulk ceramic layer in the middle of NCN‐LLZTO has stronger mechanical strength than that of the NET‐LLZTO, which can bear more tensile force transferred from the nanowire layer during the calcination process.^[^ ^45^, ^46^ ^]^ The average hardness and young's modulus of the bulk LLZTO layer with PEO filtration determined by nanoindentation are 3.19 and 75.5 GPa, respectively (Figure S8, Supporting Information). Besides, when assembling NCN electrolyte with electrodes, a corrugated structure would be formed, which endows the cell with better buffering property when a high load is applied. The successfully constructed NCN skeleton is expected to provide rapid and uniform lithium‐ion transport. Figure S1a–c, Supporting Information, exhibits the photographs of ceramic precursors of ceramic bulk sheet and electrospun nanowire‐network membrane, as well as the NCN skeleton with different layers before sintering together. The as‐obtained 3D ceramic framework with NCN structure in a large scale is also displayed (Figure S1d, Supporting Information). The above LLZTO ceramic skeletons and the cross section of NCN‐LLZTO were further characterized by the mapping of different elements (Figure S2, Supporting Information). Obviously, all the elements are distributed uniformly in the skeleton. Combined with XRD result that is given later, it also demonstrates that tantalum is successfully doped into LLZO to stabilize the cubic phase of garnet at such a low sintering temperature. To investigate the crystallization temperature of LLZTO precursor, the thermogravimetric analysis (TGA) was employed. As shown in Figure S3, Supporting Information, the TGA curve of representative NCN precursor membrane reveals that the transformation of the precursor to oxide could start at 700 °C, since no obvious weight loss is observed after this temperature.^[^ ^47^, ^48^ ^]^ After sintering the ceramic skeleton at 700 °C for 3 h, the formation of cubic Li_6.5_La_3_Zr_1.5_Ta_0.5_O_12_ (JCPDS #45‐0109) is demonstrated by XRD measurement (Figure S4, Supporting Information). The distinct peaks of cubic garnet in the composite electrolyte elucidates that incorporating PEO matrix does not have adverse effect on the active inorganic skeleton. Not only that, combing with the XRD patterns of PEO and SPE, as well as the later DSC result, the PEO matrix in the composite solid electrolyte is completely amorphous, which is beneficial for lithium‐ion transfer in the polymer region.

**Figure 3:**
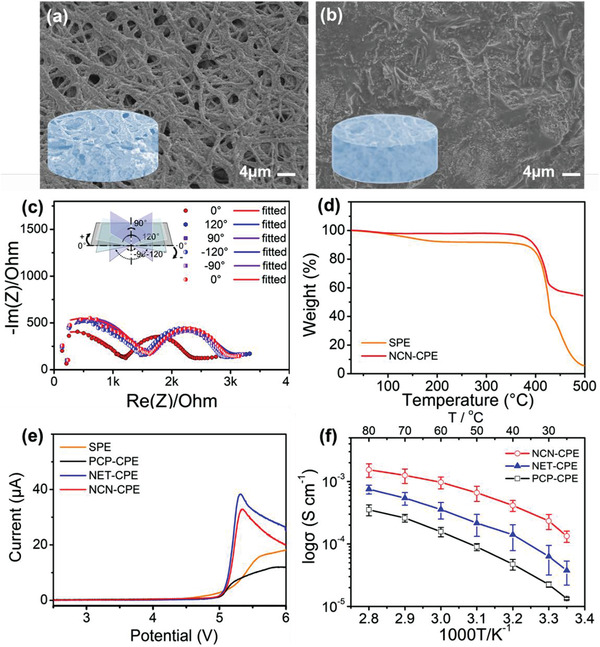
The surface morphology of NCN‐CPE (a) before h (d) after heating treatment; (c) The impedance spectra of LiFePO_4_/NCN‐CPE/Li pouch cell at various bending angles; d) TGA curves of SPE and NCN‐CPE; e) LSV curves of the SPE and CPEs; f) The Arrhenius plot of CPEs at different temperatures from 25 to 80 °C.

Figure S5, Supporting Information, exhibits the morphologies of as‐obtained composite electrolytes. A flat and smooth surface is shown for PCP‐CPE due to the good film‐forming property of PEO layer (Figure S5a, Supporting Information). In contrast, a relatively rough surface is generated for both NET‐CPE and NCN‐CPE electrolytes, which is caused by the porous structure of NET skeleton and uneven deposition of PEO during the preparation (Figure S5b,c, Supporting Information). From the cross‐sectional view of each electrolyte in Figure S5d–f, Supporting Information and the enlarged view of bulk ceramic layer in Figure S7, Supporting Information, the complete filling of polymer component and a thickness of around 200 µm for the electrolytes are displayed, demonstrating the successful preparation of the designed composite electrolytes. Since the rough surface would lead to the insufficient physical contact between electrolyte and electrode, a heating treatment was applied to eliminate this phenomenon. Compared with the original filled NCN‐CPE and NET‐CPE, a flatter surface is shown after heating the electrolyte at 80 °C for 1 h (**Figure** **3**a,b and Figure S6, Supporting Information). The addition of polymer electrolyte is not only conducive to the physical contact between electrode and NCN‐CPE, but also could act as a buffer to protect the ceramic network from being crushed under pressure. The interfacial resistance of the electrolyte with electrodes was demonstrated by assembling with LiFePO_4_ cathode and lithium foil and an equivalent circuit model used for data fitting (Figure S9, Supporting Information).^[^ ^49^, ^50^ ^]^ As shown in Figure S10, Supporting Information, before treating the pouch cell at 80 °C, the interfacial impedance of the NCN‐CPE pouch cell is larger than 20 000 Ohm. In sharp contrast, a much smaller value of 833 Ohm is observed after heating (Figure 3c), which is resulted from the surface flatness and enhanced wettability of the electrolyte on electrode surface during this process. When the NCN‐CPE pouch cell was bent into different angles for impedance testing, the battery impedance did not change significantly except the slight increase after initial bending. The good flexibility is probably ascribed to the buffer effect of corrugated structure constructed by the nanofiber network layer and PEO infiltration. An NCN‐CPE at bending state is shown in Figure S11 and Video S2, Supporting Information. From the TGA analysis in Figure 3d and Figure S12, Supporting Information, the inorganic content for NCN‐CPE, NET‐CPE, and PCP‐CPE are 58.2%, 39.2%, and 16.4%, respectively. The larger proportion of the LLZTO component is conducive to the high thermal stability and wide electrochemical window of the composite electrolyte, owing to the flame resistance and high voltage stability of active ceramics.^[^ ^2^, ^51^ ^]^ Thus, a decomposition temperature is emerged for NCN‐CPE until ≈400 °C, which endows it with good thermal stability. It is noted that the slight weight loss of PCP‐CPE at the temperature below 150 °C is probably due to the evaporation of moisture. The electrochemical properties of the 3D CPEs were characterized by an American Princeton electrochemical impedance spectroscopy. The electrochemical stability of the CPEs is shown in Figure 3e using linear sweep voltammetry (LSV). The electrochemical windows of all the prepared PCP‐CPE, NET‐CPE and NCN‐CPE can reach 5 V, while the SPE only exhibits an electrochemical stability of 4.1 V, which is improved by the high content of ceramic additions, suggesting the compatibility of designed composite electrolytes with high voltage cathodes. From Figure 3f, among the 3D composite solid electrolytes, NCN‐CPE exhibits the highest ionic conductivity of 4.15 × 10^−4^ S·cm^−1^ at 40 °C, which is almost one order of magnitude higher than that of PCP‐CPE (4.74 × 10^−5^ S·cm^−1^) and NET‐CPE (1.41 × 10^−4^ S·cm^−1^). The detailed ionic conductivities of each CPE at various temperatures are displayed in Table S1. This proves that NCN ceramic skeleton indeed has more rapid and continuous lithium‐ion conduction channels than the other two structures.

### 2.2 Lithium Dendrite Inhibition Mechanism

**Figure 4:**
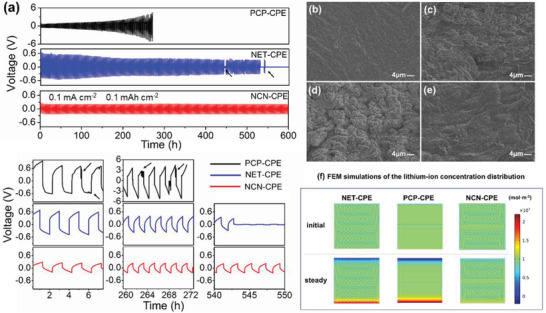
a) Li plating/stripping test with a constant current density of 0.1 mA cm^−2^ for CPEs at 40 °C; SEM morphology of b) the uncycled lithium electrode and cycled Li anode surface for c) PCP‐CPE, d) NET‐CPE and e) NCN‐CPE; f) FEM of the lithium ion density distribution for PCP‐CPE, NET‐CPE, and NCN‐CPE at the initial and stable state.

To verify the advantage of 3D‐CPEs in the cycling stability with lithium metal anode, the assembled symmetric lithium batteries (Li/3D‐CPEs/Li) were cycled using Li plating/stripping test. As shown in **Figure** **4**a, although the Li/PCP‐CPE/Li has better contact between the electrolyte and electrode, the symmetric cell displays gradually increased overpotential with the increasing test time and finally leads to the disconnection of electrode with electrode (reminded as “over voltage protection > 5 V” during the test). The phenomenon is caused by the growth of lithium dendrites and insufficient mechanical strength of polymer layer initially, followed by the blocking of bulk ceramic layer in PCP‐CPE. Notably, the relatively higher ionic conductivity of NET‐CPE contributes to the stable cycle for a period of time. However, the uneven deposition of Li^+^ on Li anode and inadequate mechanical strength caused a short‐circuit for real mode of Li/NET‐CPE/Li cell after ≈540 h. On the contrary, with the rapid and homogeneous Li‐ion transport and anion fixation in NCN structure, the Li/NCN‐CPE/Li kept cycling steadily for 600 h with barely increased overpotential. The impedance plot of the symmetric Li cell at 40 °C is displayed in Figure S13, Supporting Information, and the detailed Li plating/stripping curves are shown in the enlarged images in Figure 4a and Figure S14, Supporting Information. The two peaks behavior of Li/PCP‐CPE/Li in the initial cycles is ascribed to the repeated growth and consumption of lithium dendrites, revealing the serious concentration polarization in the electrolyte. As the cycle continues, an arcing voltage profile appeared, which could be attributed to the formation of SEI and “dead Li” on Li surface.^[^ ^52^ ^]^ In sharp contrast, Li/NCN‐CPE/Li exhibits much more flat voltage profile, indicating the more homogenous lithium concentration gradient. The not perfectly horizontal voltage profile of NCN‐CPE is probably resulted from the formation of passivate SEI film by the decomposition of PEO and lithium salts.^[^ ^53^, ^54^, ^55^ ^]^ The smallest overpotential of Li/NCN‐CPE/Li among those three CPEs demonstrates the distinct lithium conduction ability and cycling reversibility for Li plating/stripping in the electrolyte,^[^ ^56^ ^]^ as well as outstanding lithium transference number of NCN‐CPE (*t* ^+^ = 0.9, Figure S15, Supporting Information). Moreover, the Li/NCN‐CPE/Li cell was also tested with a high current density of 2 mA cm^−2^ and the areal capacity was set at 1 mAh cm^−2^, which demonstrates the good dendrite inhibition ability of designed NCN‐CPE (Figure S17, Supporting Information). We further disassembled the symmetrical Li batteries and characterized the surface morphology of the lithium anode. Obviously, a flat and smooth surface is shown for Li anode before cycling in Figure 4b. In comparison, the rough surface with lithium dendrite nucleation is appeared for both the cells using PCP‐CPE and NET‐CPE (Figure 4c,d). Nevertheless, the lithium metal anode matched with NCN‐CPE has no obvious dendrites, thereby further proving that the NCN‐structure can inhibit the formation of lithium dendrite and promote interfacial compatibility (Figure 4e). Figure S18, Supporting Information, displays the digital photographs of the metallic lithium anodes after cycling with the designed CPEs, in which the anode with PCP‐CPE has lost the metal luster, which is probably caused by reacting with moisture residue in the electrolyte as mentioned in TGA analysis.

The general approaches for designing composite electrolytes including improving their mechanical strength and incorporating soft polymer electrolyte are only able to mechanically inhibit the penetration of lithium dendrites and alleviate the rigid interfacial contact. However, it is still a challenge to fundamentally solve the formation of lithium dendrites governed by the redistribution of Li^+^ and the development of polarization effect at the interface. The detrimental Li‐ion concentration polarization would be generated and disrupts the uniform Li deposition on electrode, thereby leading to the growth of lithium dendrites. Based on this, we further elucidate the influence mechanism of our designed electrolyte structures on Li‐ions distribution at the interface using finite element modeling (FEM) simulation. Figure 4f shows the normalized lithium‐ion concentrations in the composite electrolytes, where the top is lithium metal anode and the bottom is cathode. In the pristine state, lithium ions are homogeneously distributed throughout the CPEs. Normally, the rate of Li‐ion reduction on the electrode is much faster than that of Li‐ion conduction in the electrolyte. Therefore, when a constant current was applied, the obvious Li^+^ concentration polarization at anode interface was exhibited for both the cells using NET‐CPE and PCP‐CPE, due to their relatively low ionic conductivities and anion transfer in the electrolytes. In sharp contrast, the well‐designed NCN‐CPE with rapid and uniform Li ion transport channels as well as superior anion immobilization ability displays the compressed concentration polarization when the Li‐ion concentration reaches equilibrium. The simulation results reveal that our tactic is rational and can get to the root of local Li nucleus problem on the lithium anode.

**Figure 5:**
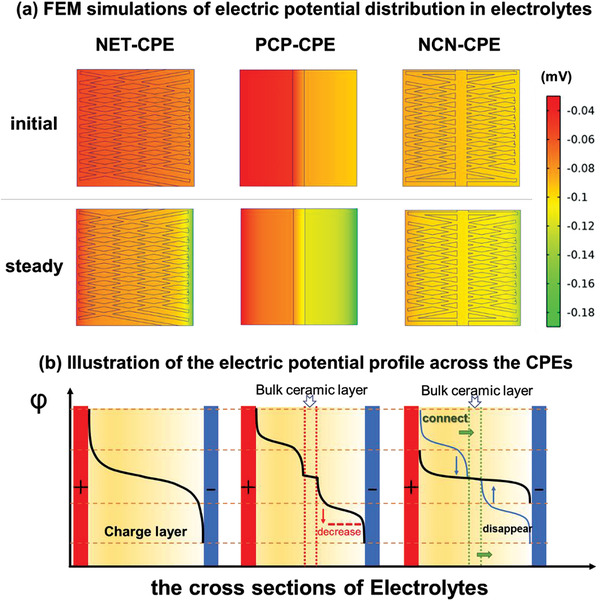
a) FEM simulations of electric potential distribution in NET‐CPE, PCP‐CPE, and NCN‐CPE; b) the illustration diagram of the electric potential profile across the CPEs for NET‐CPE, PCP‐CPE, and NCN‐CPE respectively according to the FEM simulations.

The electric potential profile at the interface of each composite electrolyte/electrode was simultaneously investigated via FEM simulations and the regions built at the interface that are different from the internal potential distribution of electrolytes (**Figure** **5**a).^[^ ^12^ ^]^ The depleted ion concentration at the electrolyte side induces the large electric field, thus, the thick space charge layers are developed for NET‐CPE and PCP‐CPE. The higher potential height of local space charge suggests a higher diffusion barrier for lithium‐ion transport and would result in the non‐uniform Li^+^ flux on the electrode. Notably, the Li‐ion deficient region at the NCN‐CPE/electrode interface can be significantly restrained owing to the adjusted interface charge carrier distribution via homogeneous and rapid ion immigration channels in the designed NCN‐CPE. The dynamic simulation results of the ion concentration and electric potential distributions are shown in Video S1, Supporting Information. A relative comparison of the electric potential profile across the solid composite electrolytes is displayed in Figure 5b, where the x‐axis is the thickness of the CPE and y‐axis is the potential height. Obviously, compared with NET‐CPE, with the addition of bulk ceramic layer in the PCP‐CPE, the electric field at the electrolyte/anode interface is reduced as indicated with a red arrow, revealing the redistribution of charge carriers at the interface with the anion blocking effect of internal ceramic layer in PCP‐CPE. Further, with the incorporation of the in situ growth of ceramic networks on bulk ceramic layer in the NCN skeleton, the differential potential is consequently disappeared at the conducting bulk ceramic/network layer interface, owing to the rapid and continuous Li^+^ transport pathways originated from the integrated NCN framework.

**Figure 6:**
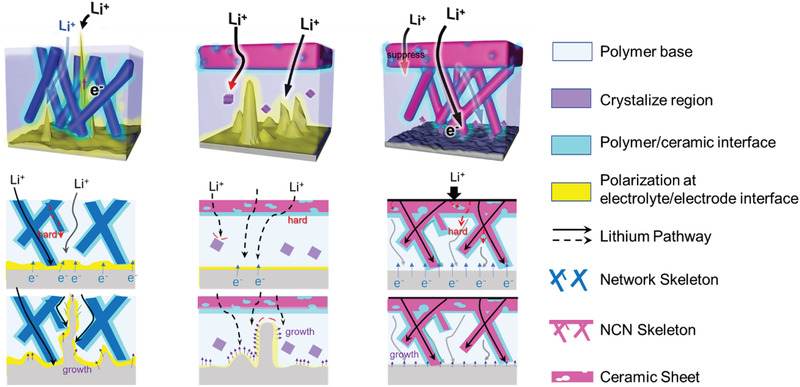
The schematic illustration of the lithium dendrite growth for the three 3D‐CPEs.

Combined with the above experimental and simulation results, a schematic mechanism illustrating the lithium dendrite growth in the cells employing the designed CPEs is exhibited in **Figure** **6**. According to the literature, Li ions in such composite electrolytes can be conducted via the Li^+^ conducting ceramic component, polymer electrolyte, and the inorganic/organic interfaces.^[^ ^57^, ^58^, ^59^ ^]^ The NET‐CPE is supposed to possess homogeneous Li^+^ flux, however, in real situation, the conducting nanowires in NET‐CPE would be fractured during the preparation process, the continuous and even Li conduction cannot be realized. More importantly, the polarization effect at the electrolyte/electrode interface would break the electrical neutrality on the lithium anode and thereby lead to the non‐uniform Li dendrite nucleation. Compared with that of NET‐CPE (Figure S16, Supporting Information), the inserted bulk ceramic with high Li transference number can block the anion of polymer salt (Figure S15, Supporting Information), and reduce the trapped positive charge at the anode‐polymer interface. Since the ceramic layer prepared in PCP structure is very thin and the polymer on both sides of the ceramic still accounts for a larger proportion of the electrolyte, therefore, only depend on ceramic layer to suppress and weaken the polarization is very limited. The increasing overpotential of PCP‐CPE in the cycling process of symmetric Li cell indicates the continuously enhanced interfacial resistance (Figure 4a). Combing with the surface morphology of Li metal anode (Figure 4b), the large interfacial impedance is probably caused by the lithium dendrites, which is detrimental to the long lifespan of the solid‐state battery. In sharp contrast, the rational designed NCN‐CPE with rapid and homogeneous Li^+^ immigration channels are expected to eliminate the formation of disadvantageous potential difference at the interface and induce the uniform Li deposition on lithium anode.

### 2.3 Battery Cycle Stability and Safety Performance

**Figure 7:**
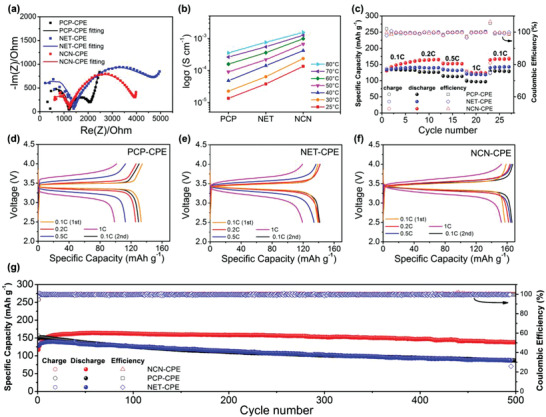
a) The impedance spectra of LiFePO_4_/CPEs/Li cells at 25 °C; b) Conductivity comparison of CPEs at different temperatures; c) Rate performance of LiFePO_4_/CPEs/Li batteries at 40 °C and d–f) their corresponding charge/discharge curves; g) the long‐cycle performance of LiFePO_4_/CPEs/Li batteries at 0.2 C and 40 °C.

The impedance spectra of the assembled LiFePO_4_/NCN‐CPE/Li, LiFePO_4_/PCP‐CPE/Li, LiFePO_4_/NET‐CPE/Li batteries at room temperature are shown in **Figure** **7**a, in which the PCP‐CPE battery possesses the smallest interface impedance, proving that the physical contact of polymer with electrode is the best. Although NCN‐CPE and NET‐CPE have the similar interfacial structure toward electrodes, the interface impedance of NCN‐CPE is still 814 Ohm smaller than that of NET‐CPE, conveying that the NCN‐LLZTO skeleton has the function of promoting ion transport and weakening the polarization at the interface, which is consistent with above analyses. According to the ionic conductivities of obtained CPEs from 25 to 80 °C in Figure 7b, the NCN‐CPE exhibits the highest ion conduction ability at various temperatures. To further determine the advantage of integrated NCN‐skeleton in battery performance, a comparison of the cycle performance employing NET‐CPE and NCN‐CPE is tested in Figure S19a–c, Supporting Information. The capacity of NCN‐CPE cell decreases slightly as the temperature decreases, while the NET‐CPE cell has an obvious capacity attenuation when the temperature is cooled to 40 °C. At high temperature, the polymer matrix can cooperate with the ceramic framework to keep the lithium‐ion transport continuously and rapidly, thus there is no significant difference of the cycle performance at 60 and 50 °C. From the DSC analysis (Figure S20, Supporting Information), the glass transition temperature (TG) of pure PEO electrolyte is around −43 °C and the TG of polymer matrix is further decreased after reinforced with NCN skeleton, revealing that polymer chains are more relaxed in NCN‐CPE, which endows it with better wettability toward electrodes. Besides, the corrugated 3D skeleton of NCN‐LLZTO is able to keep its integration and continuous rapid Li^+^ conduction pathways, while the nanowire structure is easier to be interrupted during the preparation process. Thus, the larger polarization potential of NET‐CPE battery shown in Figure S19c, Supporting Information, especially at lower temperature, is probably ascribed to the slow Li^+^ immigration in the electrolyte and consequently developed concentration polarization at electrolyte/electrode interface.

The adverse effect of the polarization effect at the interface would result in the inferior rate performance of the cells. As displayed in Figure 7c–f, the battery with NCN‐CPE delivers the specific capacity of 157.3, 163.4, 152.2, 124.4 mAh·g^−1^ at 0.1, 0.2, 0.5, 1 C, respectively, which is superior to other two electrolytes. By contrast, the PCP‐CPE cell with large polarization exhibits the worst rate performance, even though it possesses better physical contact with electrodes among the designed three CPEs. All the batteries can recover to their initial capacities when the current density return back to 0.1 C. The long‐cycle stability of the LiFePO_4_/CPEs/Li was further demonstrated at 0.2 C and 40 °C (Figure 7g). The NCN‐CPE cell displays an initial discharge capacity of 116.9 mAh g^−1^ and 117.5% (with respect to the first cycle) as well as 90.2% capacity retention (with respect to the highest capacity) after 500 cycles. In contrast, the capacities only maintained 86.5 mAh g^−1^ and 87.3 mAh g^−1^ for the cells with PCP‐CPE and NET‐CPE, respectively. The outstanding cycling performance of NCN‐CPE cell is resulted from the combined action of its high ionic conductivity and excellent electrolyte/electrode interface stability. Moreover, the cycling stability of obtained CPEs was also tested at a higher current rate of 1C (Figure S19d, Supporting Information), and a similar good performance is emerged for NCN‐CPE cell. A pouch cell of LiFePO_4_/NCN‐CPE/Li was further assembled and cycled at 0.2 C and 40 °C. As seen from Figure S21, Supporting Information, an initial discharge capacity of 131.2 mAh g^−1^ is obtained for the pouch cell and still retain the capacity of 102.9 mAh g^−1^ after 50 cycles (78.4% capacity retention). Moreover, the cycling performance of our designed electrolyte assembled with NCM811 was also evaluated from 2.7 to 4.3 V at various rates in Figure S22, Supporting Information. The stable cycle of solid‐state NCM811/NCN‐CPE/Li cell demonstrates that the designed electrolyte is compatible with high voltage cathode.

**Figure 8:**
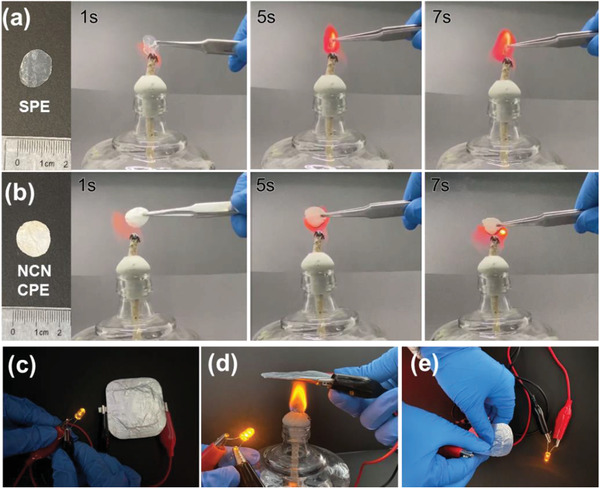
Flame resistance test of a) solid PEO electrolyte (SPE) and b) NCN‐CPE; c) LiFePO_4_/NCN‐CPE/Li pouch cell lighting up an LED lamp at room temperature; Safety performance of LiFePO_4_/NCN‐CPE/Li pouch cell by heating and bending test.

The flame retardancy of the solid electrolytes are shown in **Figure** **8**a,b. The pure polymer electrolyte (SPE) deformed and burned immediately after being placed on the flame, and completely disappeared after 7 s. On the contrary, NCN‐CPE remained its intact structure after the same burning time. The good flame resistance of NCN‐CPE is attributed to the large ceramic ratio in the composite, which is thereby beneficial to improve the battery safety. Figure 8c shows an LED lamp is lightened up by our assembled punch cell using NCN‐CPE. After treating with heating and bending process (Figure 8d,e), the LED bulb is still lighted, revealing the good safety performance and flexibility of the designed composite electrolyte.

## 3 Conclusion

In this work, we designed a special ceramic skeleton with 3D indented structure, where the nanowire‐networks are in situ growth on both sides of a bulk ceramic sheet, forming an integrated nanowires‐bulk ceramic‐nanowires (NCN) structure. By means of the finite element analysis, we found that the homogeneous and continuous Li‐ion immigration channels, as well as polarization effect at electrolyte/electrode interface, are optimized by adjusting the ceramic framework structure for the first time. Our NCN‐structure battery displays stable electrochemical performance in the bending state, and superior long‐term stability in 0.2 and 1 C. The electrochemical window of NCN‐CPE can reach 5 V, and the ionic conductivity at 40 °C is 4.15 × 10^−4^ S·cm^−1^. The battery with NCN‐CPE delivers an initial capacity of 116.9 mAh g^−1^ and a capacity retention as high as 117.5% after 500 cycles is achieved. In addition, NCN‐CPE can cycle stably for 600 h in the lithium plating/striping test at a current density of 0.1 mA cm^−2^, showing excellent interface stability and lithium dendrites suppression ability. This work not only ensures the high ion conduction structure, but also further improves the cycle stability of the battery through interface optimization, which proposes a new direction for interface optimization for the future construction of 3D ceramic skeleton reinforced composite electrolytes.

## 4 Experimental Section

The experimental method and related characterization are listed in the supporting documents.

## Conflict of Interest

The authors declare no conflict of interest.

## Supporting information

Supporting Information

Supplemental Video 1

Supplemental Video 2

## Data Availability

Research data are not shared.
