# Supplementary material for: Regulating Interfacial Li‐Ion Transport via an Integrated Corrugated 3D Skeleton in Solid Composite Electrolyte for All‐Solid‐State Lithium Metal Batteries
Source: Adv Sci (Weinh). 2022 Jan 17;9(8):2104506. doi: 10.1002/advs.202104506 (PMC8922129; doi:10.1002/advs.202104506)
Supplement: Supplementary file 1 — Supporting Information [file ADVS-9-2104506-s003.pdf]

ADVANCED SCIENCE
Open Access

Supporting Information

for *Adv. Sci.*, DOI 10.1002/advs.202104506

Regulating Interfacial Li-Ion Transport via an Integrated Corrugated 3D Skeleton in Solid Composite Electrolyte for All-Solid-State Lithium Metal Batteries

*Rong Fan, Wenchao Liao, Shuangxian Fan, Dazhu Chen, Jiaoning Tang, Yong Yang and Chen Liu**

ADVANCED SCIENCE
Open Access

Supporting Information

for *Adv. Sci.,* DOI: 10.1002/advs.202104506

Regulating Interfacial Li-ion Transport via an Integrated Corrugated 3D Skeleton in Solid Composite Electrolyte for All-Solid-State Lithium Metal Batteries

Rong Fan, Wenchao Liao, Shuangxian Fan, Dazhu Chen, Jiaoning Tang, Chen Liu and Yong Yang*

Supporting Information

Regulating Interfacial Li-ion Transport via an Integrated Corrugated 3D Skeleton in Solid Composite Electrolyte for All-Solid-State Lithium Metal Batteries

Rong Fan, Wenchao Liao, Shuangxian Fan, Dazhu Chen, Jiaoning Tang, Chen Liu and Yong Yang*

Experimental Section

Preparation of ceramic skeletons

Preparation of LLZTO thin ceramic sheet

$Li_{6.5}La_3Zr_{1.5}Ta_{0.5}O_{12}$ (LLZTO) ceramic sheet was prepared by sol-gel method. First, dimethylformamide (DMF) and acetic were mixed at a volume ratio of 5:1. Then lithium nitrate ($LiNO_3$, ≥ 99 %), lanthanum nitrate hexahydrate ($La(NO_3)_3{\cdot}6H_2O$, ≥ 99 %), zirconium propoxide ($ZrC_{12}H_{28}O_4$, 70 wt % in 1-propanal), tantalum ethoxide ($C_{10}H_{25}O_5Ta$, ≥ 99 %) were added by a molar ratio of 7.475:3:1.5:0.5, in which 15 % excess $LiNO_3$ used to compensate for Li loss during calcination. After the salt was fully dissolved, 4 wt% polyvinyl pyrrolidone (PVP, Mw = 1300000) was added in the mixture and stirred overnight to obtain the LLZTO gel solution. Afterwards, the solution was casted on a smooth aluminum foil to form a gel film. The film was then placed in a vacuum oven at 80 °C for 6 h to evaporate the solvents. Finally, the LLZTO ceramic sheet was obtained by calcining at 700 °C for 3 h at a rate of 1 °C min^{-1}.

Preparation of LLZTO network skeleton (NET-LLZTO)

NET-LLZTO was prepared by electrospinning process. Firstly, the sol-gel solution of LLZTO was prepared as same as the LLZTO ceramic sheet, and then electrospuned at the speed of 0.05-0.08 mL·min^{-1} under a voltage of 15~20 kV and collected on the aluminum foil. Finally, the nanofiber film was peeled off from the foil and calcined at 700 °C (1 °C min^{-1}) for 3 h in the air and LLZTO network skeleton was attained after the annealing process.

Preparation of the nanowire-bulk ceramic-nanowire LLZTO skeleton (NCN-LLZTO)

The preparation of NCN-LLZTO skeleton combined the sol-gel method and electrospinning method. First, the gel film and nanofiber film were prepared according to the above-mentioned methods. Then the gel film was sandwiched between two nanofiber membranes and rolling pressed together. The films should be kept flat during the rolling process to avoid air bubbles and the gel film cannot be completely dry Thereafter, the prepared NCN-LLZTO precursor membrane was put in a muffle furnace at a rate of 1 °C min^{-1} and sintered at 700 °C for 3 h. The 3D nanowires-bulk ceramic-nanowires (NCN) skeleton was finally obtained.

Preparation of 3D composite electrolytes

The composite electrolytes were prepared via solution-casting method. For PEO-LiTFSI polymer matrix, PEO (Mn = 600000, Sigma-Aldrich) and lithium bis(trifluoromethane) sulfonimide (LiTFSI, Sigma-Aldrich) were dissolved in acetonitrile with an EO: Li^+ molar ratio of 8: 1 and kept stirring for 3 h. The polymer solution was casted on a Teflon mould and subsequently dried overnight under vacuum at 60 °C to remove the solvent. The prepared films were dried in a glove box

for further use. For the polymer-ceramic-polymer structure composite electrolyte (PCP-CPE), the prepared LLZTO sheet was placed between two polymer films and rolling pressed together through a laminator. Taking care that the gas needs to be removed completely. For the 3D network structure composite electrolyte (NET-CPE) and integrate NCN-CPE, the active ceramic skeletons also coated the polymer matrix by solution-cast method. The ceramic skeletons were soaking in a small amount of polymer solution at first and dried in a vacuum oven for 2 h at room temperature and 12 h at 60 °C to remove the solvent. This process needs to be repeated several times until the ceramic was fully coated by the polymer. An extra heating process at 80 °C for 1 h under pressure was employed if the surface of CPEs is not flat. Finally, the obtained composite electrolyte membranes were stored in an argon-filled glove box with H_2O and O_2 contents below 0.1 ppm to completely evaporate the solvent residue before use.

Characterization of 3D Structure Composite Electrolytes

X-ray diffraction patterns (XRD) of the LLZTO skeleton and NCN-CPE were examined by Brucker AXS D8 diffractometer with Cu Kα radiation (40 kV and 300 mA) in the 2θ range of 10°~70°, λ=1.5418Å. The morphologies of the LLZTO skeletons and three-dimension composite electrolytes were characterized by a field-emission scanning electron microscope (FESEM, Hitachi SU-70) with energy dispersive X-ray spectroscopy (EDS). Differential scanning calorimetry (DSC) measurements were conducted on a TA instrument (DSC 25) with a heating rate of 10 °C min^{-1} from -80 °C to 120 °C under N_2 atmosphere. The thermogravimetric

analysis (TGA 55, TA instruments) was conducted under an air atmosphere with a heating rate of 10 °C min^{-1} to determine the formation temperature of LLZTO ceramic skeleton for the calcination process.

Electrochemical properties of obtained composite electrolytes

The cells were all assembled in a glove box and tested by an American Princeton electrochemical impedance spectroscopy (EIS, PMC-1000). The ionic conductivity of the CPE was measured between two stainless steel (SS) blocking electrodes in a frequency range of 3 MHz to 0.5 Hz ranging from 25 °C to 80 °C. Electrochemical stability and cycling performance measurements were conducted by assembling Li/CPEs/SS cells and symmetrical Li cells, respectively. The electrochemical stability was determined by linear sweep voltammetry (LSV) at a scan rate of 0.5 mV s^{-1} from open circuit voltage to 6 V. The lithium ion transference number was determined by Bruce-Vincent method. A constant DC bias (0.01V) was applied to the symmetric Li/ CPE/Li cells and the initial current value i_0 was measured until a steady current i_s was reached after 1-4 h. The equation for the calculation of cationic transference number t_{Li^+} was as follows: $t_{Li^+} = \frac{i_s\left(\Delta V - i_0 R_0^{film}\right)}{i_0\left(\Delta V - i_s R_s^{film}\right)}$. The values of initial and steady state are represented by subscripts 0 and s, respectively, R^{film} represents the passive film resistance, ΔV is the applied voltage, and i stands for the current. All the resistance values were acquired from the impedance spectra carried out in the frequency range of 3 MHz and 1 Hz. The lithium plating/stripping cycling performance was determined at at 40 °C. The all-solid-state lithium batteries were

assembled by using $LiFePO_4$ or $LiNi_{0.8}Co_{0.1}Mn_{0.1}O_2$ (NCM811) as the cathode and lithium metal as anode. The cathode mass loading was maintained at around 1.2 mg cm^{-2}. The cycling performance of the batteries were monitored by a LAND CT2001A instrument. For the pouch cell assembly, the preparation process of electrodes and composite electrolytes was similar to that for coin cells. An electrode welding operation was employed to bonding the solid electrolyte. Afterwards, the cell was sealed in an aluminum plastic package using a vacuum hot-pressing machine in glove box.

Finite element analysis (FEM) Simulation

Finite element analysis was employed to monitor the lithium-ion concentration and potential distribution of the three different structure electrolytes by COMSOL Multiphysics. A 20 mV voltage was applied to the upper surface and simulated 30 min for the lithium-ion distribution and potential distribution calculation. In the calculation, three calculation models are used as follow: 1) First model is composed of polymer PEO and LLZTO ceramic layer and the middle LLZTO ceramic layer is 20 µm. 2) The second model is set to be LLZTO nanowire network; 3) The third model is the structure of LLZTO nanowires grown on bulk ceramic sheet, where the thickness of the middle ceramic sheet is 20 µm. The thickness of all the models is set to be 200 µm and the length is set to be 800 µm. In our work, the three models are denoted as PCP-CPE, NET-CPE and NCN-CPE. The migration of Li ions and free anions driven by electric field and diffusion flow were considered in simulations. Two physical models of electrostatic and transport of diluted species based on the partial

differential equations listed below were coupled to conduct FEM simulation:

$$E = -\nabla\psi$$

$$N = -D\nabla = c + ucE$$

$$\frac{\partial c}{\partial t} = -\nabla N$$

where ψ is the electric potential, E is the electric field, D is the diffusion coefficient of Li ion, c is the concentration of Li ion, m is the ionic mobility of Li ion in electrolytes, and N is the flux vector of Li ion. These FEM simulations on the routine, our composite electrolyte was performed in a rectangle area, respectively. The potential difference Dϕ through these electrolytes is set at 20 mV. To investigate the ion transport behaviors with limited electrolytes in long time cycling, the same physical model was established and the ratio of diffusion coefficients of Li ions in polymer electrolytes and ceramic electrolytes was decreased to 10.0. The mobilities of Li ions m for polymer electrolyte and ceramic electrolytes are defined by the Nernst-Einstein equation. The bottom boundaries of two simulation areas are the Dirichlet boundaries with ϕ0 = 0 V and c0 = 0 M. The top boundaries of two simulation area are also Dirichlet boundaries with ϕ1 = 0.02 V and c1 = 1.0 M. The other boundaries are natural boundaries with zero flux.

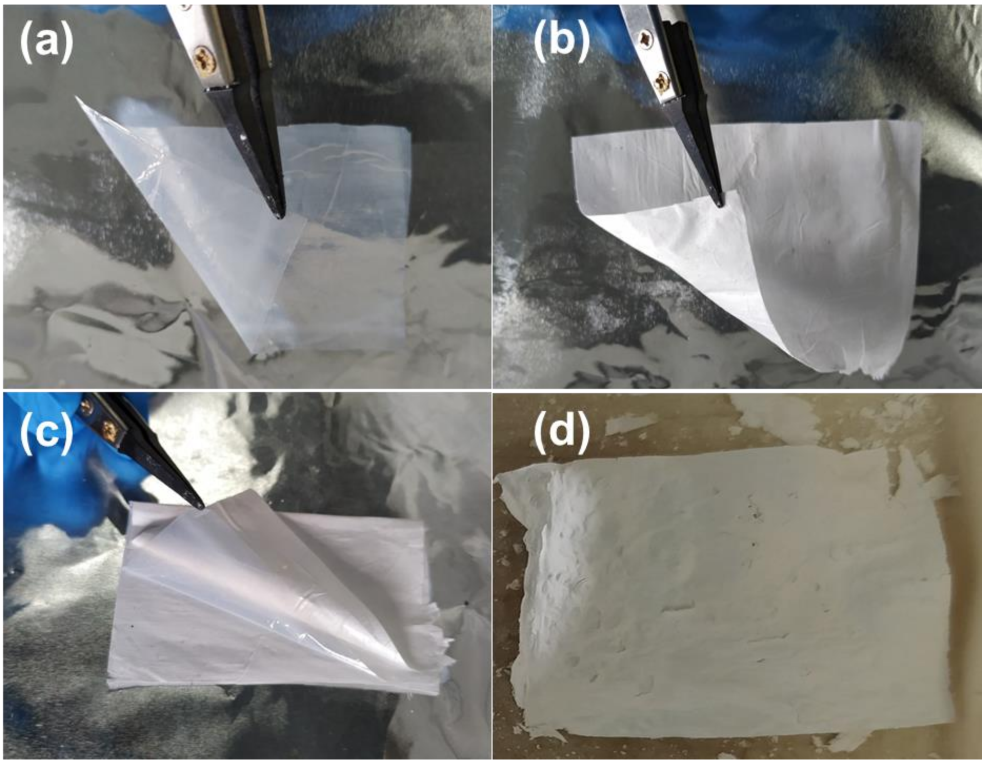

Figure S1. Photographs of ceramic precursors of (a) the obtained sol-gel film of bulk ceramic sheet, (b) electrospun nanowire-network membrane, the NCN skeleton (c) before and (d) after sintering.

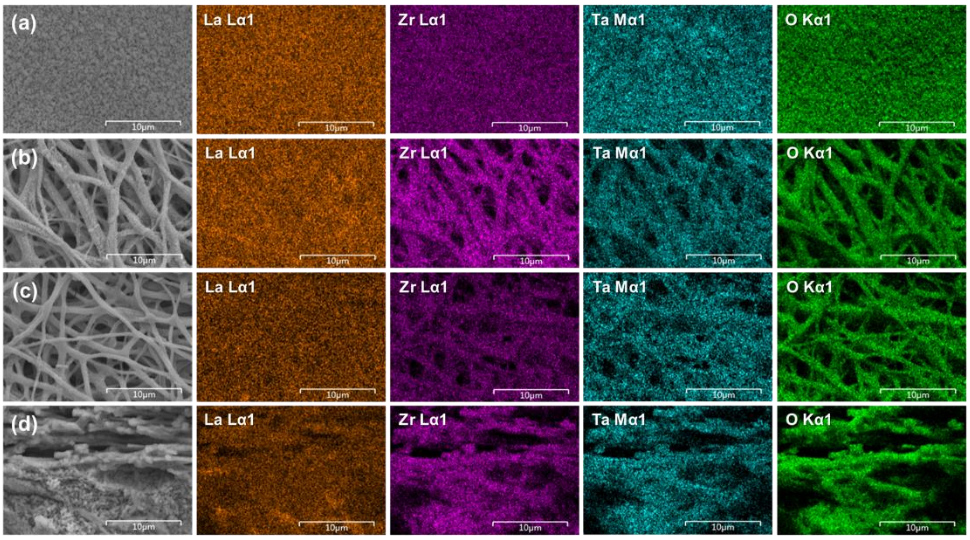

Figure S2. The EDS mapping images of the active ceramic skeleton: (a) the surface of the LLZTO bulk ceramic, (b) the surface of the LLZTO ceramic network, (c)&(d) the surface and cross section of the NCN ceramic skeleton.

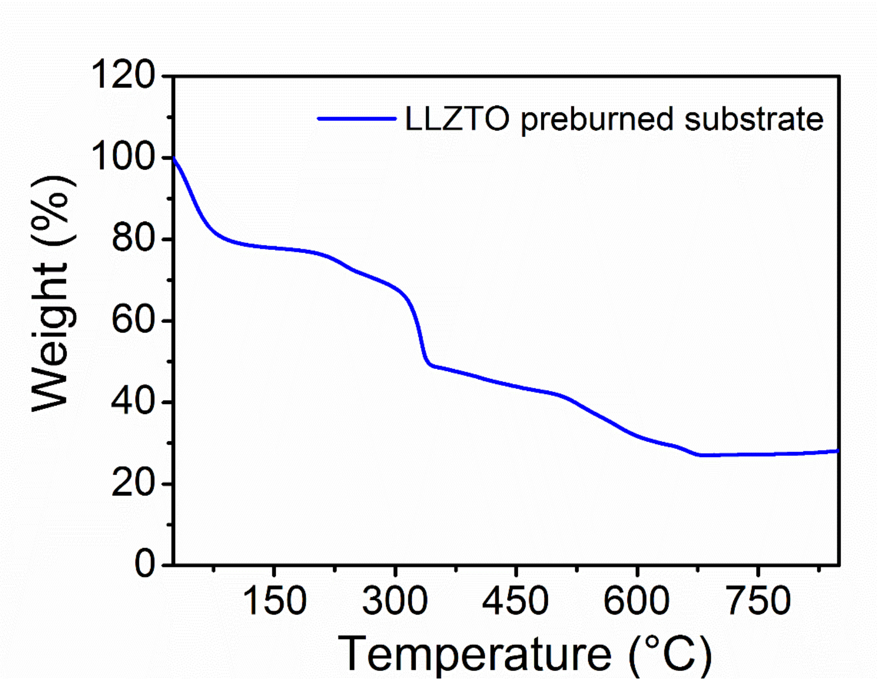

Figure S3. TGA curve of the NCN precursor membrane.

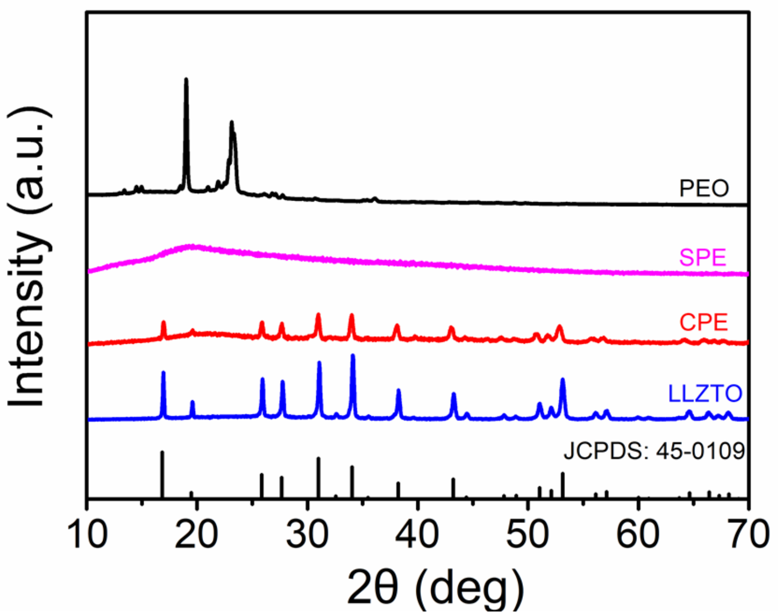

Figure S4. XRD curves of PEO, SPE, NCN-LLZTO skeleton and NCN-CPE.

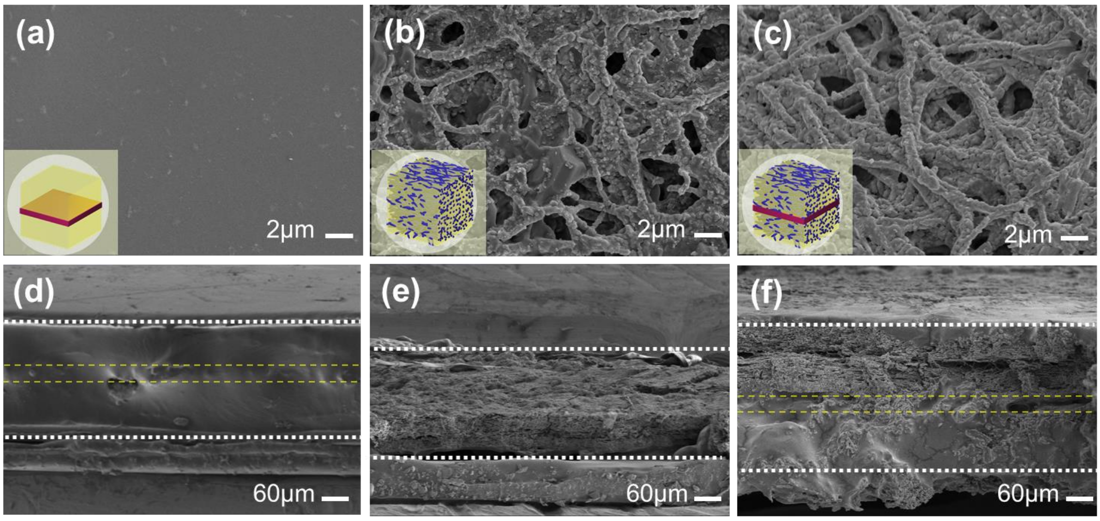

Figure S5. SEM images of solid composite electrolytes: (a)-(c) top-view and (d)-(f) the cross-sectional view of PCP-CPE, NET-CPE and NCN-CPE electrolyte before heat-treatment, respectively (the area between the dotted yellow lines is the bulk ceramic layer).

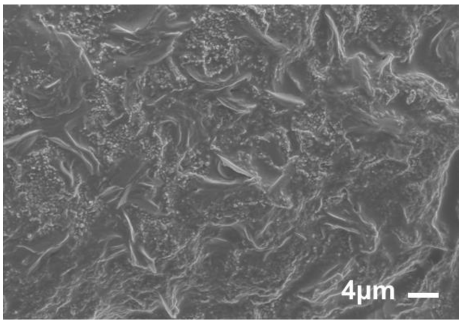

Figure S6. SEM image of the top view of NET-CPE after heat-treatment.

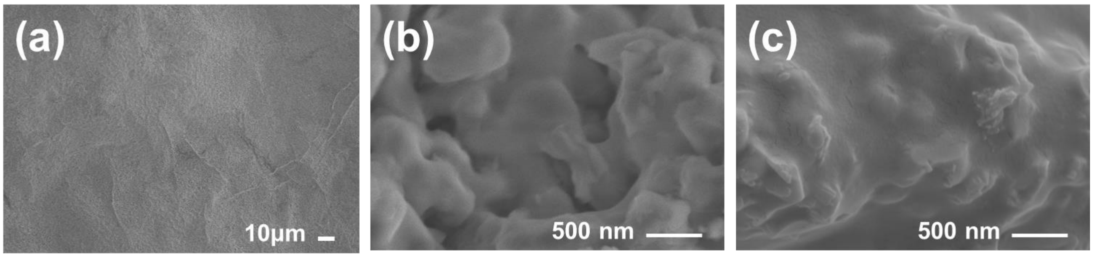

Figure S7. (a) Top view of bulk ceramic layer; Enlarged cross-sectional view of (b) bulk ceramic layer and (c) its corresponding infiltration of PEO.

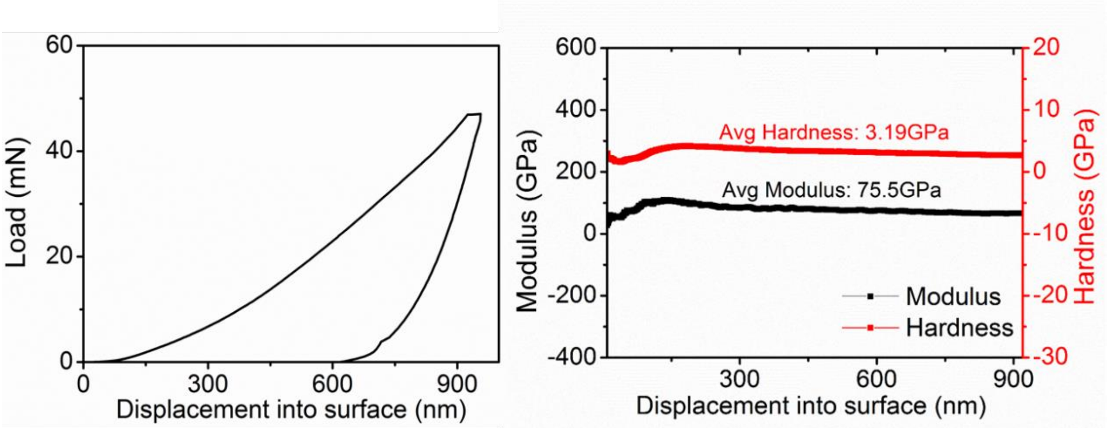

Figure S8. Load displacement curve (left) and the average hardness, young's modulus of bulk LLZTO layer with PEO.

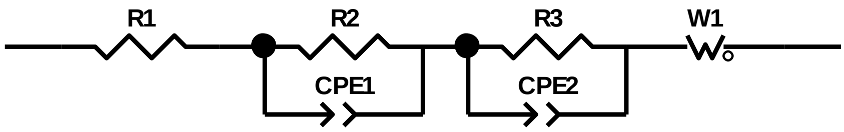

Figure S9. Equivalent circuit used for EIS data fitting.

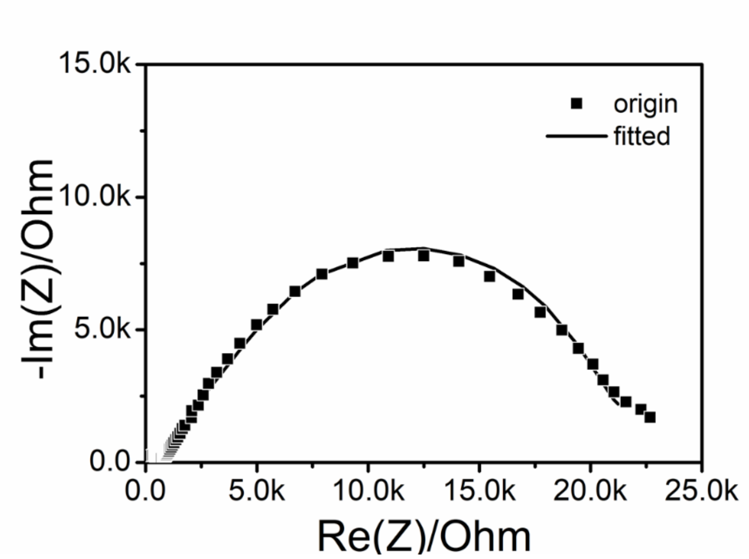

Figure S10. Impedance spectrum of original $LiFePO_4$/NCN-CPE/Li pouch cell before heating treatment.

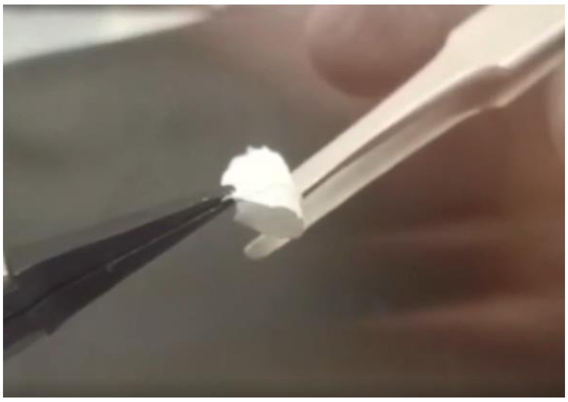

Figure S11. Photograph of NCN-CPE at bending state.

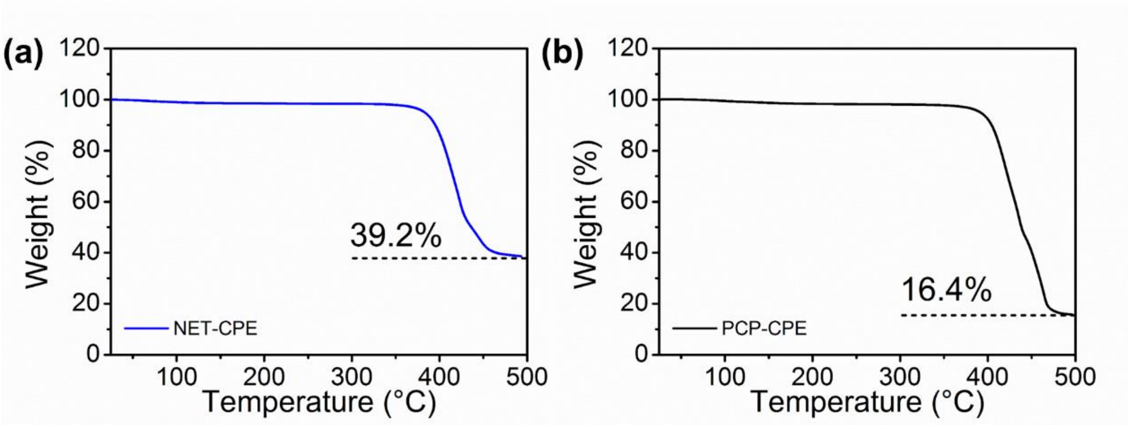

Figure S12. TGA curves of NET-CPE and PCP-CPE.

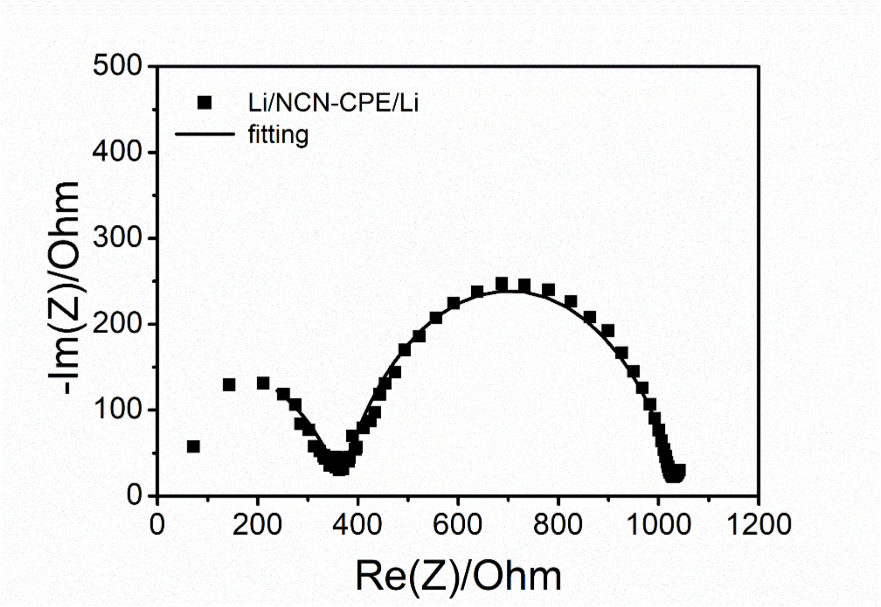

Figure S13. The impedance plot of Li/NCN-CPE/Li at 40 °C for the cell using in Li plating/stripping test at 0.1 mA cm^{-2}.

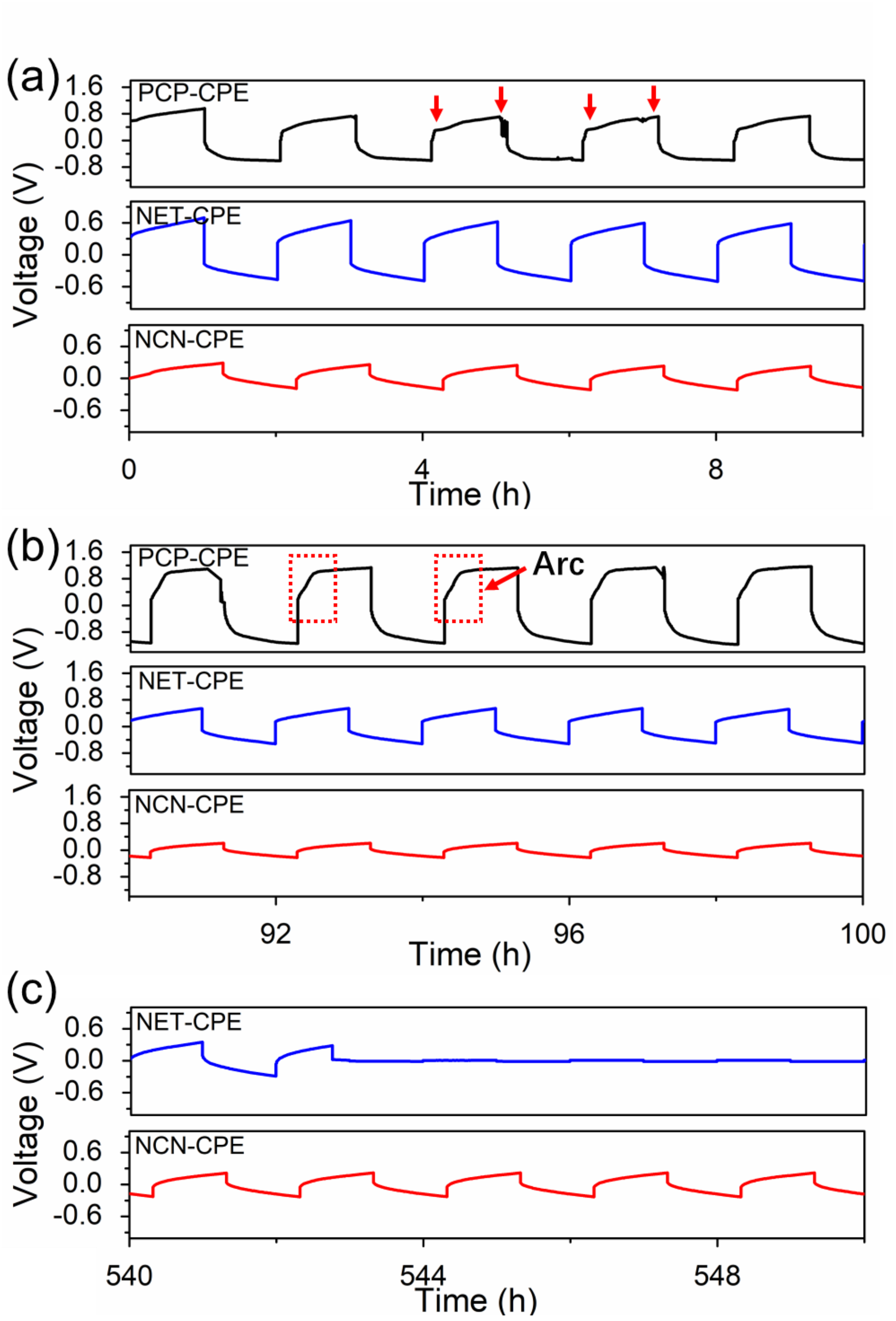

Figure S14. Partial enlarged detail of Li plating/stripping test with a constant current density of 0.1 mA cm^{-2} for CPEs at 40 °C.

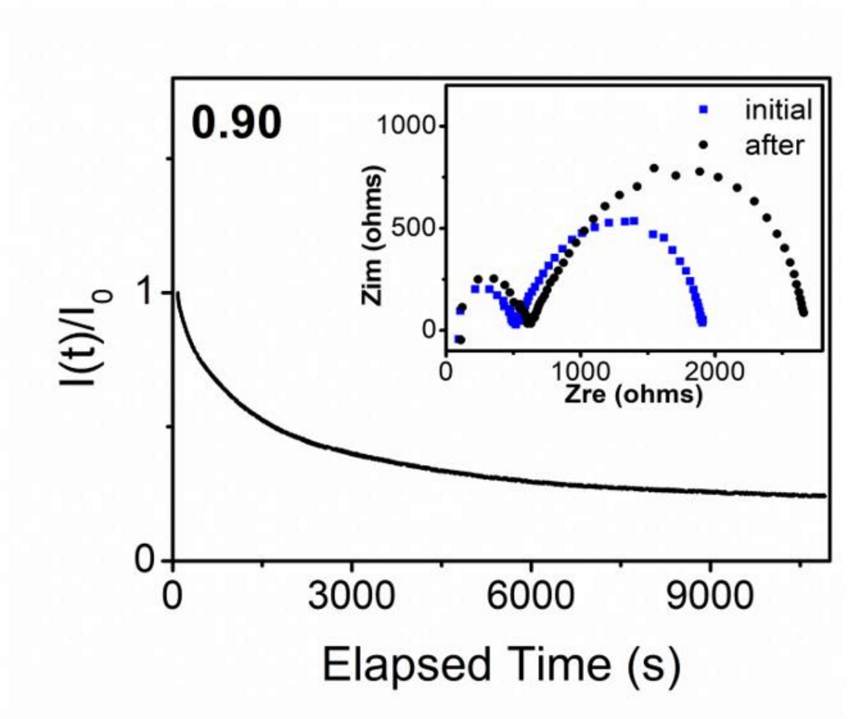

Figure S15. Li ion transference number of NCN-CPE.

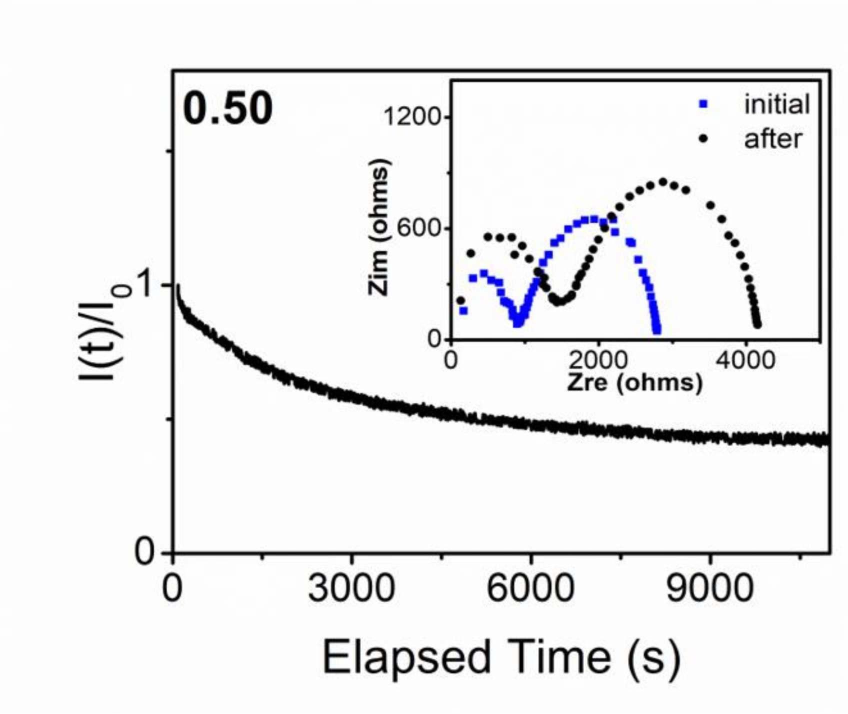

Figure S16. Li ion transference number of NET-CPE.

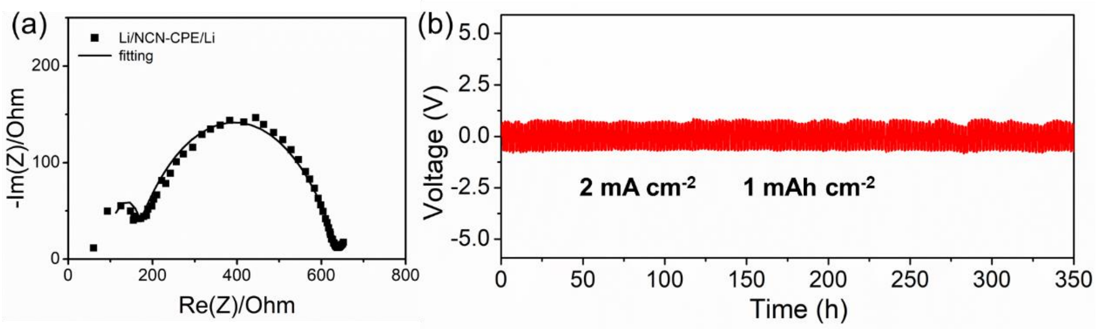

Figure S17. (a) The impedance plot of Li/NCN-CPE/Li at 40 °C for the cell using in Li plating/stripping test at 2 mA cm^{-2}, (b) Li plating/stripping test for NCN-CPE at 2 mA cm^{-2} and 40 °C.

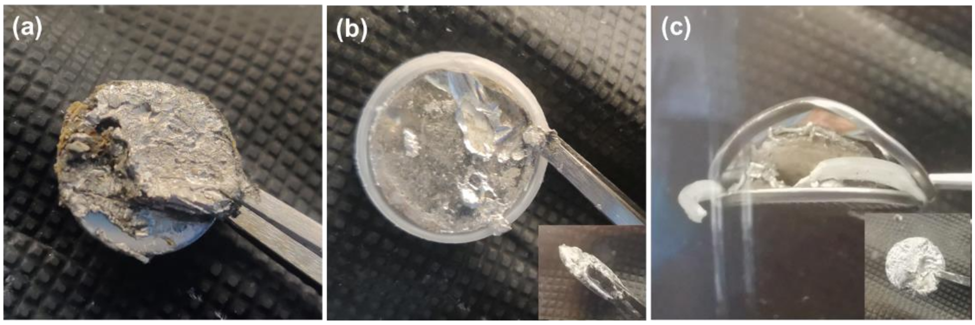

Figure S18. Photographs of the lithium anodes for (a) PCP-CPE, (b) NET-CPE and (c) NCN-CPE after cycle.

Table S1. The lithium-ion conductivities for the obtained CPEs (S cm^{-1})

	25°C	30°C	40°C	50°C	60°C	70°C	80°C
NCN-CPE	1.34×10^{-4}	2.34×10^{-4}	4.15×10^{-4}	6.60×10^{-4}	9.71×10^{-4}	1.25×10^{-3}	1.54×10^{-3}
NET-CPE	3.77×10^{-5}	6.33×10^{-5}	1.41×10^{-4}	2.16×10^{-4}	3.57×10^{-4}	5.44×10^{-4}	7.52×10^{-4}
PCP-CPE	1.34×10^{-5}	2.24×10^{-5}	4.74×10^{-5}	8.98×10^{-5}	1.57×10^{-4}	2.61×10^{-4}	3.52×10^{-4}

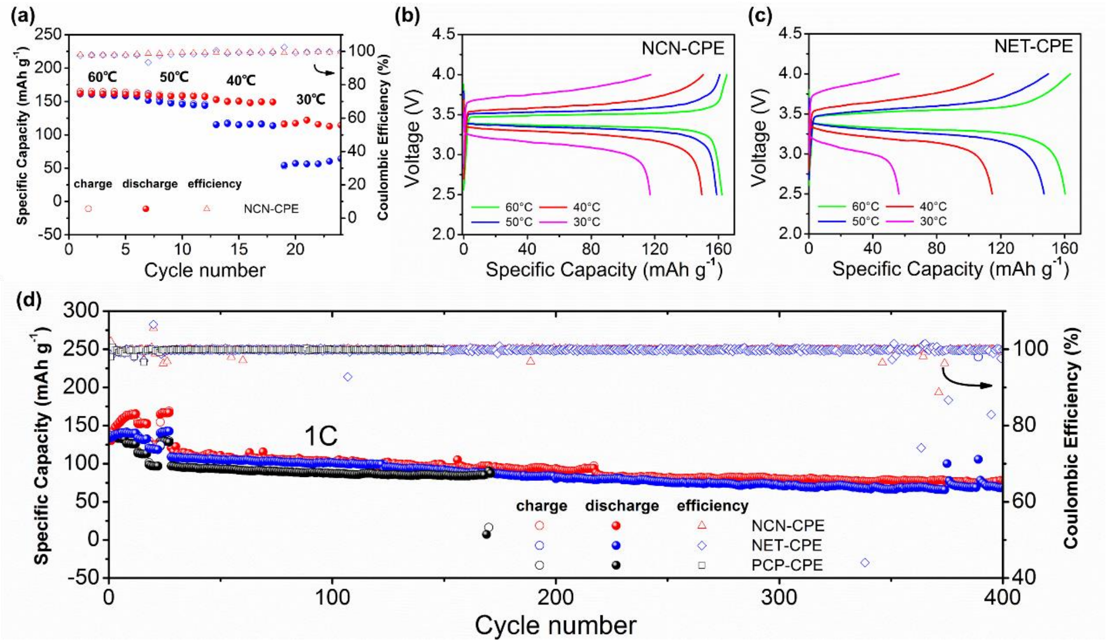

Figure S19. (a) Cycle performance of $LiFePO_4$/CPEs/Li batteries at 0.2 C under various temperatures and (b,c) the corresponding charge/discharge curves, (e) Long cycle performance of $LiFePO_4$/CPEs/Li cells at 1 C and 40 °C.

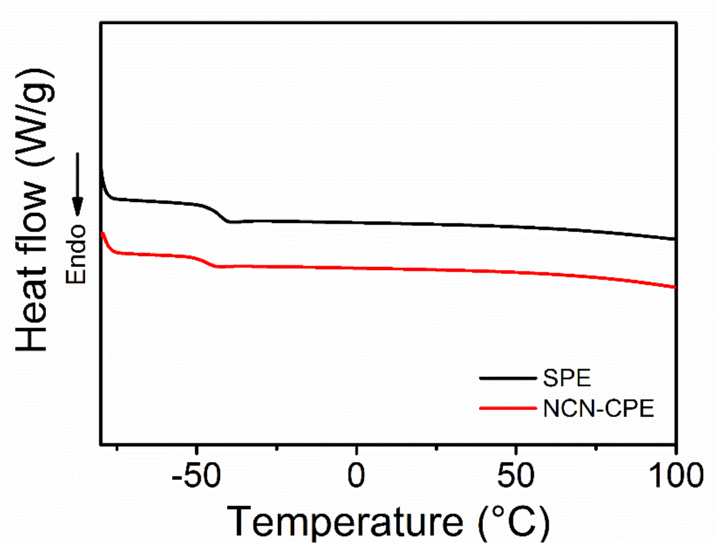

Figure S20. DSC curves of the solid polymer electrolyte (PEO_8(LiTFSI)) and NCN-CPE.

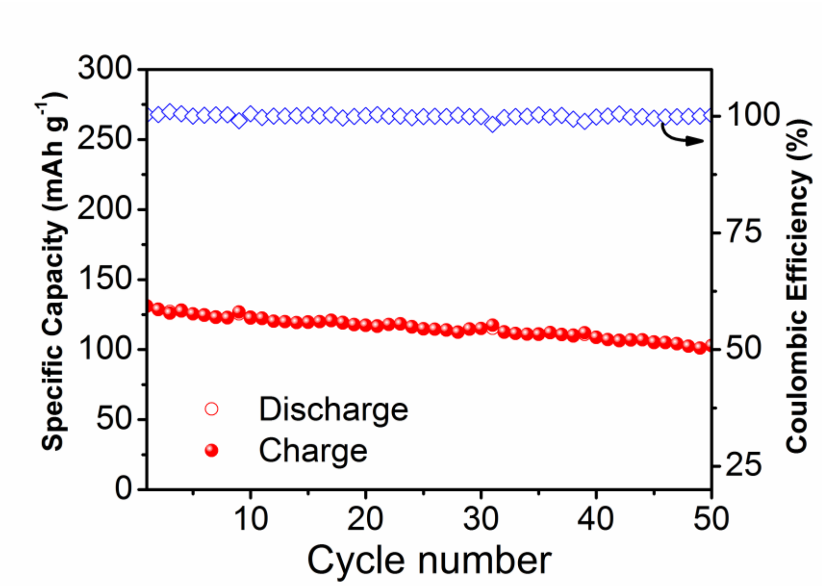

Figure S21. Cycle performance of $LiFePO_4$/NCN-CPE/Li pouch cell at 40 °C and 0.2 C.

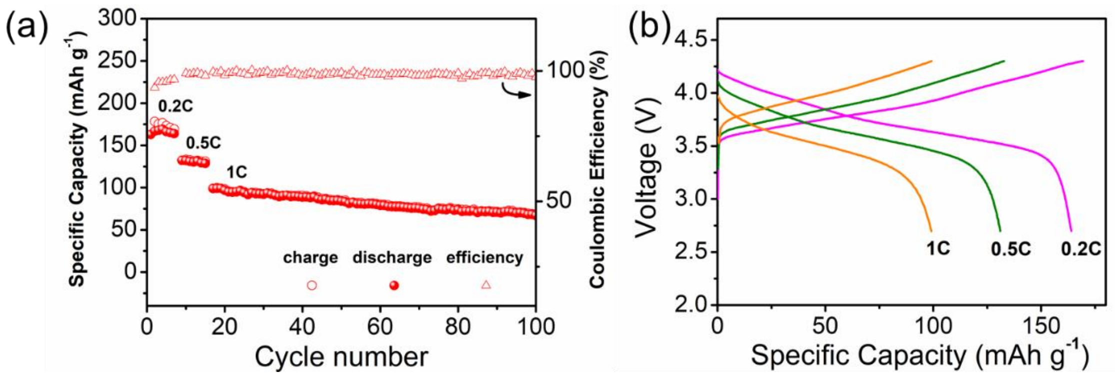

Figure S22. Cycle performance of NCM811/NCN-CPE/Li cell at various rates.
